# Health facility committees and facility management - exploring the nature and depth of their roles in Coast Province, Kenya

**DOI:** 10.1186/1472-6963-11-229

**Published:** 2011-09-22

**Authors:** Catherine Goodman, Antony Opwora, Margaret Kabare, Sassy Molyneux

**Affiliations:** 1 Kenya Medical Research Institute - Wellcome Trust Research Programme, P.O. Box 43640 - 00100, Nairobi, Kenya; 2 London School of Hygiene & Tropical Medicine, Keppel St. London WC1E 7HT, UK; 3 Impact Research & Development Organization, P.O Box 9171, Kisumu, Kenya; 4 Kenya Medical Research Institute - Wellcome Trust Research Programme, P.O. Box 230, Kilifi, Kenya; 5 Centre for Tropical Medicine, University of Oxford, OX3 7LJ, UK

## Abstract

**Background:**

Community participation has been emphasized internationally as a way of enhancing accountability, as well as a means to enhance health goals in terms of coverage, access and effective utilization. In rural health facilities in Kenya, initiatives to increase community accountability have focused on Health Facility Committees (HFCs). In Coast Province the role of HFCs has been expanded with the introduction of direct funding of rural facilities. We explored the nature and depth of managerial engagement of HFCs at the facility level in two rural districts in this Coastal setting, and how this has contributed to community accountability

**Methods:**

We conducted structured interviews with the health worker in-charge and with patients in 30 health centres and dispensaries. These data were supplemented with in-depth interviews with district managers, and with health workers and HFC members in 12 health centres and dispensaries. In-depth interviews with health workers and HFC members included a participatory exercise to stimulate discussion of the nature and depth of their roles in facility management.

**Results:**

HFCs were generally functioning well and played an important role in facility operations. The breadth and depth of engagement had reportedly increased after the introduction of direct funding of health facilities which allowed HFCs to manage their own budgets. Although relations with facility staff were generally good, some mistrust was expressed between HFC members and health workers, and between HFC members and the broader community, partially reflecting a lack of clarity in HFC roles. Moreover, over half of exit interviewees were not aware of the HFC's existence. Women and less well-educated respondents were particularly unlikely to know about the HFC.

**Conclusions:**

There is potential for HFCs to play an active and important role in health facility management, particularly where they have control over some facility level resources. However, to optimise their contribution, efforts are needed to improve their training, clarify their roles, and improve engagement with the wider community.

## Background

Community accountability can be defined as listening to and responding to the views and inputs of the public, citizens, or users, and is increasingly being emphasized in health delivery in developing countries [1-3]. A range of mechanisms have been introduced to strengthen community accountability at the facility level, including health facility committees (HFCs), patients' rights charters, suggestion boxes, customer care desks and health clubs. At the level of peripheral health facilities such as dispensaries, health centres and health posts, local committees are the most widely documented mechanism, with some information available on their implementation or impact from a wide range of countries including Kenya, Uganda, Tanzania, Niger, Nigeria, Benin, Zambia, South Africa, Peru, Mexico, Cambodia and Nepal (Molyneux et al.: Community accountability at peripheral health facilities, submitted).

Although some notable successes have been documented in committee operation (e.g. [4-6]), a number of significant challenges have also been identified. These have included problems with the selection and functioning of committees, lack of clarity in roles and responsibilities, difficulty in sustaining voluntary membership over time, insufficient resources, and inadequate representation of and links with the wider community (Molyneux et al.: Community accountability at peripheral health facilities, submitted). However, the evidence base remains limited, with only a handful of studies documenting in detail how community accountability mechanisms have operated or influenced health services, and how their performance can be improved. The literature is particularly limited in its analysis of the depth of community involvement, which may vary from mere information provision to consultation, representation, and at the greatest depth, actual influence over decisions [7-10].

This paper examines the nature and depth of community accountability through HFCs in Coast Province, Kenya, focusing on their role in facility management. HFCs in Coast Province have been relatively well supported and have responsibility for a component of the facility budget, so there is the potential for committees to overcome some of problems identified in other settings.

The Kenyan Government officially established HFCs in 1998 [11], although in some facilities similar community-based or NGO supported mechanisms existed before then. The official roles of HFCs are described in Table 1[12]. In Coast Province HFCs were strengthened through management training provided through the Ministry of Health and Danish International Development Agency (DANIDA) Health Services Project [13]. In 2005 HFC roles were expanded in Coast to include the management of a budget under the Direct Facility Financing (DFF) scheme, which was piloted in this province. DFF is an innovative finance mechanism to provide additional funds at facility level. It was introduced in response to a reduction in user fee levels in 2004, which significantly reduced facility income, and was argued to have undermined the relationship between committee members and communities ([14,15]).

**Table 1:** Roles and Powers of Kenyan Health Facility Committees

Roles of the HFC	
1	To oversee the general operations and management of the health facility

2	To advise the community on matters related to the promotion of health services

3	To represent and articulate community interests on matters pertaining to health in local development forums

4	To facilitate a feedback process to the community pertaining to the operations and management of the health facility

5	To implement community decisions pertaining to their own health

6	To mobilise community resources towards the development of health services within the area

**Powers of the HFC**	

1	The committee shall have the authority to raise funds from within itself, the community or from donors and other well-wishers for the purpose of financing the operations and maintenance of the facility

2	The committee shall have authority to hire and fire subordinate staff employed by itself in the health facility

3	The committee shall oversee the development and expansion and maintenance of the physical facilities within their respective area

Source: Managing a Health Facility: A Handbook for Committee Members and Facility Staff. Ministry of Health & Aga Khan Health Service, Kenya, Second Edition, 2005 [13]

DFF funds were transferred directly into facility bank accounts, with public health centres and dispensaries receiving an average of USD 3,392 per year [16]. The Government continued to cover the vast majority of facility resources through in kind provision of infrastructure, trained health workers, supervision, drug kits and medical supplies. HFCs budgeted for the DFF funds within specific guidelines, implemented work plans and kept accounts. Funds could be spent on a wide variety of items including wages for support staff; maintenance of buildings, furniture and equipment; travel allowances; stationary; fuel and non-drug medical supplies. The wage bill could not exceed 30% of disbursed funds, and expenditure on HFC allowances and drugs was not allowed. DFF supervision was provided by the District Health Management Team (DHMT), with the team members most involved being the facility management nurse (FMN) and the district accountant. Additional oversight was provided by two provincial level accountants. The post of FMN was created to support links between facilities, the community and the district by strengthening the management of committees. This involved overseeing the selection of committee members, organizing training, and assisting committees in planning and continuously evaluating those plans.

DFF in theory has the potential to strengthen community accountability as it involves additional HFC training, additional support through the FMN, and provides HFCs with control of some resources. Details of the implementation and effects of DFF in general are described elsewhere [16]. This paper assesses the nature and depth of managerial engagement of HFCs at the facility level, and how this has contributed to community accountability in the context of the DFF financing mechanism. We investigate HFC characteristics and training, the perceived breadth and depth of HFC managerial roles, and the relationships between HFC members and both health workers and the wider community.

## Methods

Data were collected between October 2007 and March 2008, 2 to 3 years after the introduction of DFF. Two of the 7 districts in Coast Province were purposively sampled to reflect diversity of experience with DFF implementation according to managerial views. Kwale, which was viewed as a relatively strong performer, is close to the provincial headquarters (Mombasa) and comparatively accessible. Tana River, which was perceived to have experienced more problems with DFF implementation, is 5 hours' drive from Mombasa and suffers from poor roads and infrastructure.

The sampling frame included all government health centres and dispensaries with at least one qualified health worker. Structured interviews were conducted with the health worker in-charge in a sample of 15 facilities in each district, including all health centres (5 in Kwale and 4 in Tana River), and a random selection of dispensaries (10 of the 47 eligible in Kwale, and 11 of the 25 eligible in Tana River). Health workers were asked about the facility, the functioning of the HFCs and about other community engagement mechanisms. At the same facilities we conducted structured exit interviews with a target sample of 10 community members seeking outpatient curative services per facility, obtaining a total of 292 completed questionnaires. Data were collected on patient characteristics and awareness of community engagement strategies.

A subset of 6 facilities from each district was re-visited for in-depth individual interviews with the health worker in-charge, and group discussions with a representative range of HFC members. The 6 facilities were purposively selected to include only those where the in-charge had been in post for at least 1 year, and to encompass variation in facility type, accessibility and indicators from the structured survey. Between one and nine HFC members participated in group discussions, often including the chair and treasurer. Finally, 7 in-depth interviews were conducted with members of the DHMTs: two District Medical Officers of Health (DMOHs), two FMNs, one District Health Administrative Officer (DHAO), one district health accountant and one provincial facility grants accountant.

In-depth interviews with health workers and HFC members included an exercise to stimulate discussion of the nature and depth of their roles in facility management tasks. To develop the exercise, we drew on frameworks that distinguish between depths of community involvement from simple information sharing with communities or their representatives, consultation with these groups, or - suggesting most depth of involvement - communities or their representatives having a tangible influence on health policy or practice [1,9]. A number of managerial tasks were identified, including employment of professional health workers; employment of support staff; setting the level of user fees; deciding how DFF funds are spent; deciding how user fee funds are spent; and disciplining health workers. Six cards, each with one of these managerial tasks, were given out in turn and respondents were asked to discuss what they thought their roles were regarding the task. In each situation, respondents were asked to decide whether their role entailed (1) making the final decision, (2) being consulted but with the final decision made by someone else, or (3) having no role at all. We deliberately selected situations where we expected committee members to have a variety of roles based on government guidelines (see Table 1). These included some activities where we expected HFCs to make the final decision (allocating DFF funds; allocating user fee revenues; employing casual workers); and some where we expected them to have no role (replacing the facility in-charge; disciplining health workers; setting user fees).

Quantitative data were double entered using Fox-pro D-base IV, MS Access or MS Excel, and imported into STATA version 9 for analysis. We used the STATA svy commands to adjust for clustering at the facility level and stratification by facility type and district, and variation in sampling probability across facilities. Notes were taken during qualitative interviews, and where possible, interviews were digitally recorded. Discussions were transcribed and imported into N-Vivo 7 for coding and analysis. A coding scheme was developed from a thematic framework and from reading a sub-set of the transcripts to identify the main themes.

Informed consent was obtained for all interviews, and the study was approved by the Ethical Review Committees of the Kenya Medical Research Institute and the London School of Hygiene and Tropical Medicine.

## Results

### HFC characteristics

All facilities surveyed had active HFCs. Their characteristics are presented in Table 2. Committee members included the health worker in-charge of the health facility as secretary and between 8 and 18 community members (median 10). The chair and the treasurer were chosen from the community members. Most of the latter were farmers, though some were professionals such as teachers, and a few were community health workers (CHWs). A few committees had Area Chiefs and Councilors as members by virtue of their official role. All committees had at least one female member as required by DFF guidelines (range 1 - 7; median 3).

**Table 2:** Characteristics of Health Facility Committees (HFCs) (n = 30)

Characteristics	Median^1 ^(Range)		
	**Dispensaries**	**Health Centres**	**All facilities**

N	21	9	30

Number of HFC members**^2 ^**	10 (8 - 18)	13 (9 - 15)	10 (8 - 18)

Number of female HFC members**^2 ^**	3 (1 - 7)	3 (1 - 6)	3 (1 - 7)

HFC allowances per meeting (2007 US$)^3^	1.47 (0 - 4.42)	2.95 (1.47 - 7.37)	1.47 (0 - 7.37)

Number of HFC members trained in facility management and financing**^2^**	3 (0 - 13)	2 (0 - 5)	3 (0 - 13)

Number of Staff members trained in facility management and financing	1 (0 - 2)	0 (0 - 2)	1 (0 - 2)

HFC tenure (years)	3 (1 - 5)	3 (2 - 3)	3 (1 - 5)

Number of HFC meetings held in the last quarter	2 (0 - 8)	2 (1 - 3)	2 (0 - 8)

Number of HFC members**^2 ^**present in last meeting	8 (4 - 14)	11 (7 - 15)	8 (4 - 15)

Source: Structured interviews with health facility in-charge

^1 ^Weighted to account for variation in sampling probability across facilities.

^2 ^Refers to community members of the HFC only, excluding the health worker in charge who acted as secretary

**^3 ^**Converted using the average USD/KES exchange rate for 2007 (1USD = 67.82KES). Source: http://www.oanda.com/currency/historical-rates

All HFCs had a constitution which outlined rules and codes of conduct regulating committee functioning, such as frequency of meetings. At 24 of the 30 facilities the committee had met in the preceding quarter, with a median of 2 meetings per committee (range 0 - 8), and a median of 8 members (range 4 - 15) attending the last meeting. In all cases, minutes of the last meeting were available. Minor decisions were made by smaller executive committees, consisting of the chairman, treasurer and secretary. In 27 facilities HFC members were reported to receive a sitting allowance ranging from USD 0.74 - 7.37 per meeting, funded from user fee revenues; at the remaining 3 facilities it was reported that no allowances were given (Table 2).

HFC regulations required that members be elected by the community. Health workers reported that committee members were selected in two main ways. In most cases, the village headmen or chiefs convened public meetings (barazas) within the facility catchment area, where residents from each village were given the opportunity to elect a representative from their community. In a few areas, particularly in Kwale, chairmen of existing village health committees (VHCs) were automatically selected to represent their village on the HFC. VHC members in turn had been elected by village members at a public meeting presided over by the village headman. The tenure of community members ranged from 1 to 5 years (median 3) (Table 2).

Some concerns were expressed about the selection process for HFCs. For example in Tana River, a district manager stated that although electing committee members was the best way to ensure representation, this often resulted in selection of very old, often illiterate members who could not grasp key concepts or deal with management tasks.

*"...Again another challenge is that when you tell the community *[to select] *committee members, another village decides to elect a very old person who doesn't know how to write. The reasoning is vague, in fact during the training some of them were just brushing their teeth waiting for the tea break ..." *(District manager, Tana River)

Other health workers and district managers were also concerned about HFC members having little or no formal education. The DHMT in Tana River was therefore sensitizing the community on the importance of electing people with at least basic reading and writing skills. However, managers in both districts were concerned that even these were inadequate levels of education given the financial management issues they needed to cover.

*"So you can imagine teaching financial issues, all those regulations, bar lines and cash books to people who probably went to school up to say class 7, or class 6 somewhere there, it really takes long for them to grasp the concept." *(District manager, Kwale)

One option for addressing low educational levels is including the rural elite, such as retired professionals, in committees. However, one district manager said this was problematic because such members have a tendency to monopolize meetings and can fail to include other ordinary members in decision making. Ordinary members can also be intimidated by more educated members and keep a lower profile in decision making.

As a result of the generally low education levels of committee members, health workers said they felt obliged to play multiple roles within the committees, for example, training the treasurer, or taking up the treasurer's roles. This was sometimes a source of conflict between health workers and other committee members, with the latter feeling that their roles were being usurped when health workers, for example, completed cashbooks or vouchers. In several health facilities, HFC members resented this assistance, arguing that they should have been facilitated to perform the work by themselves. However, health workers said they felt obliged to assist, as the ultimate responsibility for complete documentation lay with them.

### HFC training

At least two training sessions for HFC members had been organized and facilitated by the DHMTs in both districts between 2001-2005. Due to the turnover of managers, health workers and HFC members, it was difficult to ascertain exactly what had been covered at what point. However, the first training was said to have focused on committee roles in general, while the second, conducted in preparation for DFF roll out, focused on budgeting, expenditure and book-keeping. In Tana River the DFF training was conducted at two different venues, and covered all committee members. In Kwale, the training was organized at a single venue and restricted to committee officials only (chair, treasurer and secretary), due to what managers described as insufficient funds to train all members.

Both committee members and health workers described the DFF training as successful in terms of providing basic knowledge in financial management. However, health workers complained that there were no training aids to facilitate learning, and that having to prepare DFF work plans and budgets during the 3-day training meant that the tasks were performed hurriedly and with many mistakes. One health worker also felt that there was poor interaction between facilitators and the audience, with insufficient time devoted to key issues such as definition of their roles and financial management.

*"The time was not adequate because it really needs time for everybody to grasp what is being said and this is not a one man's show so everybody should have a turn to ask questions.....who is to remain with the finances when maybe I'm on leave? And who is really to stay with the cash book, who is to stay with the finances themselves? Such kinds of things were not touched." *(Health worker, Tana River)

In Kwale, the decision to train committee officials led to some committee members feeling left out and alienated from the whole process. They suggested that in future the whole committee should be involved in all activities from planning to implementation. Health workers from both districts similarly advocated for the training of all committee members, saying it would reduce the workload of health workers and reduce suspicion between health workers and other committee members.

*"There is need for the community's participation *[through the committee] *in everything because they are the inhabitants of this area while the health worker is only here for a time and can decide to leave." *(Health worker, Tana River)

Post-training, HFCs received support from the FMNs who visited facilities frequently during the early stages of DFF implementation to monitor the functioning of committees and provide assistance to those having difficulties. However, by the time of data collection, district managers reported that most facilities were managing activities well and visits had been reduced.

*"After the trainings we did monthly follow-ups for up to 6 months. Thereafter we were doing follow-ups once every 6 months...the committee members are doing well, they are operating well, and they are implementing their activities." *(District manager, Tana River)

### Breadth and depth of HFC roles

There was consensus among interviewees that HFCs had two main roles: representation of community interests to the facility; and overseeing facility operations and management. On the former, HFC members said they brought issues raised by the community to health workers, and some health workers noted that if a problem arose in the community, such as a disease outbreak, the first people to notify them were committee members.

As far as managing the health facility was concerned, committee members felt that this was an important part of their role.

*"Eeeh . . . we really believe that at the point we are elected, we are given the mandate to oversee the daily operations of the health facility"*. (HFC Member, Tana River)

Other roles mentioned by committee members included community mobilization, defaulter tracing for patients on anti-tuberculosis drugs, and organizing health education talks in facilities. Some of the latter activities also involved CHWs. HFC members also participated in outreach clinics, which health workers held in more remote villages, by mobilizing the community and sometimes assisting health workers by for example, weighing babies and keeping records.

According to district managers in both districts, prior to implementation of DFF, HFCs were rarely active. In Tana River for example, it was reported that many committees had stopped meeting regularly, had no constitution in place, and no work-plans. Some members had resigned and those who remained had little awareness of their roles. A similar situation was reported in Kwale where HFC members were described by a district manager as being 'blank' at the time of DFF introduction.

Committee functioning was generally perceived to have improved with the introduction of DFF. This was partly attributed to the additional funds at facility level. Before DFF, committees had managed revenue from user fees only, which involved much lower sums. The majority of the interviewees felt that though DFF had not changed broad HFC roles, the scope of their activities had widened considerably since committees could plan and implement activities with the additional finances, increasing their participation and developing their sense of facility ownership.

*"You know management without finance is not management at all. Now if it couldn't be these *[DFF] *funds these committees couldn't be meeting often like that because they would have nothing to discuss about or to budget for." *(Health worker, Tana River)

DFF was also said to have improved participation through the provision of meeting allowances for HFC members. Committee members reported that before DFF, facility funds were insufficient to pay allowances, but the introduction of DFF freed up some user fee revenues for this purpose.

*"Previously, we depended on the cost sharing money only and it was too little, just enough for drugs or syringes but not allowances...members would not come for meetings because there were no allowances." *(HFC member, Tana River)

While they described their work in the facility as largely voluntary, allowances were seen as partially compensating members for the time spent on health-related activities, thus increasing commitment to facility management and improving general committee functioning. However, allowances were still viewed as insufficient in many cases, with several HFC members recommending that they be increased, or introduced where they were currently not provided.

Results of the card exercise shed light on both the scope and depth of HFC roles across a range of facility management issues. There was only one scenario with unanimous agreement, with all groups stating that HFCs made the final decision about employment of subordinate staff (Table 3). In nearly all discussions it was also agreed that HFCs made the final decision on user fee expenditure.

**Table 3:** Summary of the Results of the Card Exercise (n = 23 discussions^1^)

Scenario	Respondent	Make Final Decision	Be Consulted	No Role to play	Not decided
Employing casual staff	HFC	12	0	0	-
	
	HW	11	0	0	-

How to spend user fees	HFC	11	1	0	-
	
	HW	11	0	0	-

How to spend DFF	HFC	7	5	0	-
	
	HW	10	0	0	1

Replacement of the health worker managing the facility	HFC	0	5	7	-
	
	HW	0	4	7	-

Disciplining health workers	HFC	1	9	2	-
	
	HW	1	10	0	-

Increasing user fee charges	HFC	4	2	5	1
	
	HW	5	2	4	-

Source: In-depth interviews with HFC members and health workers

^1 ^at one dispensary the card exercise was not performed with the health worker due to lack of time

HFC = Health facility committee; HW = Health worker in charge of facility; DFF = Direct Facility Funds

Views were less clear-cut on DFF expenditure. While 10 of the 11 health workers said the HFC had the final decision here, only 7 of the 12 HFC groups agreed, with 5 saying that they were only consulted. One in-charge could not decide on where their role lay. Members held lengthy debates amongst themselves on the extent of their autonomy on this issue, taking into account the restrictions on DFF expenditure.

*"...we cannot make a fast decision on this one (*DFF expenditure.)*If we are given 20,000 then told we must spend it this way and not any other way...do we make final decisions or our role would only be to give views?... Because we do have a small role" *(HFC member, Kwale)

Moreover, the Tana River DHMT had distributed budget plans to each facility, allocating funds by expenditure category, apparently due to insufficient time during training for budget preparation, and the low level of education of some HFC members. HFCs were allowed to request alterations but this required DHMT approval, leading some to state that actual decisions on DFF expenditure were made 'there by them', referring to district managers and DANIDA staff.

*"Here *(referring to use of DFF funds), *we cannot say we give views or make the final decision because when this money comes, it's already broken down and there are guidelines of expenditure; I don't know what to say in this case..."*(HFC member, Tana River)

In Kwale, while budgets were prepared during training, only the executive committee members had attended, meaning that some ordinary members felt excluded from this responsibility. However, there was no difference in the general pattern of responses on spending DFF funds across districts.

Most groups felt the HFC had no role in employing qualified health workers, although some felt they should be consulted, A majority said committees were supposed to be consulted over disciplining health workers; HFCs could not make any final decisions but could refer their complaints to the district level.

The greatest variation in views was expressed around the HFC role in increasing user fee charges. HFCs were split among the three options of making the final decision, being consulted and having no role (Table 3), with one group failing to reach a decision. The views of health workers were also very mixed. The groups stating that the HFC had no role argued that the Ministry of Health had the mandate to set user fees levels. However, other groups felt HFCs could make changes as long as they consulted with the community before implementation. A few committees reported having increased charges in the past, with the agreement of community members and the DHMT. District managers agreed that officially only the national Ministry of Health could set user fee levels, but said that if a facility had extra needs, and the community agreed, the DHMT would allow higher fees.

### Relationships between community committee members and health workers

Overall, there was cooperation between health workers and community HFC members across both districts. A number of health workers said they valued the opportunity to discuss issues with the other HFC members and were satisfied with their level of participation in facility activities.

*"Committee members are the people who bring in information from whatever problem there is in the community. They are the ones to report it. When it's time to tackle those problems that affect the community, they are the ones to present these problems." *(Health worker, Tana River)

In one facility in Kwale, committee members said they trusted the health workers and described the working relationship between them as cordial.

*"In this committee we really trust our in charge; the in charge is very co-operative. But in other areas, this may not be the case..." *(HFC member, Kwale)

Similar views were expressed by several committee members in other facilities within the district. However, in a few facilities some areas of conflict had arisen, primarily related to HFC roles, financial management and allowances. These cases were reported in both districts, but more frequently in Tana River. Several health workers complained that since the committees had been told the facility was theirs, they had started acting like "watchdogs", trying to supervise everything the health workers were doing and undermining them. A district manager concurred with this view.

*"...after the training people hoped that there would be good co-ordination between the rural facility in-charge and the committees, but then the committees somehow thought that they were like watchdogs. So it became very difficult for some of the facility in-charges to operate and this used to cause a lot of delays till now the FMN had to go to the facilities and resolve some of these conflicts." *(District manager, Tana River)

One health worker in Tana River described committee members as stubborn, uncooperative and a nuisance and felt that too much power had been vested in them, even suggesting their abolition. In Kwale, a district manger reported that in one facility committee members had demanded money for their "personal issues" and refused to sign vouchers for facility-related expenditure until their requirements were met. The same manager reported that committee members from another facility had refused to vacate their office when their term ended.

Committee members also had some complaints. A district manager in Tana River reported that committee members in one facility accused the health worker of hiding financial information from them. In another facility in the same district, HFC members were said to have demanded that all the facility's money be withdrawn from the bank account and kept at the house of the treasurer, because of a lack of trust in the health worker in-charge.

The majority of health workers and district managers thought the major source of these conflicts was that committees either did not know their role in facility management or had misunderstood it to mean supervising the health workers instead of working together. The members' limited capacity and education may have exacerbated the conflict according to a district manager, and they stressed the need for further training to avoid such problems in future. A few health workers thought that improving communication between them and the rest of the committee would also reduce the conflict associated with misunderstanding.

### Interactions between HFC members and the wider community

On a positive note, interviewees in both districts felt that relationships between HFC members and the wider community were generally good: a district manager said that community members were happy to have representatives on HFCs, and committee members felt they represented the community with legitimacy.

However, exit interview data indicated that awareness of HFCs, and knowledge and interaction with the HFC chair or members, remained low among facility clients. Only 44.8% had ever heard of the HFC, with only 16.2% knowing the Chair, and 23.9% knowing any member (Table 4). We assessed whether there was any variation in these indicators by exit interviewee characteristics. Awareness was significantly higher in Tana River than in Kwale, among males, and among those who had attended school or were literate. Interaction with HFC members also appeared low, with only 4% of all interviewees having ever spoken to a HFC member regarding facility management issues. The most commonly raised issues were lack of drugs and health workers, and lateness of staff reporting to work.

**Table 4:** Community Members' Knowledge of HFCs

Characteristics of exit interviewees		Ever heard of HFC		Knows HFC Chair^1^		Knows any HFC Member^1^	
	**N**	**292**		**292**		**292**	

		%^2^	p-value	%^2^	p-value	%^2^	p-value

All Cases		44.8		16.2		23.9	

District	n	292		290		286	

	Kwale	34.5	0.009**	9.8	0.018**	16.0	0.007**

	Tana River	60.3		25.9		36.5	

Type of facility	n	292		290		286	

	Health Centre	37.8	0.171	10.2	0.011**	22.4	0.59

	Dispensary	45.8		17.0		24.3	

Gender	n	292		290		286	

	Male	61.1	0.0006***	33.1	0.0002**	37.9	0.004**

	Female	41.3		12.6		21.1	

Age	n	292		290		286	

	16-24	31.4	0.011**	9.7	0.146	15.7	0.016**

	25-44	49.7		20.2		30.2	

	45 & Above	54.9		17.6		23.3	

Attended School	n	289		288		284	

	Yes	54.5	0.005**	23.8	0.005**	30.3	0.044**

	No	34.1		7.7		17.3	

Can Read Kiswahili	n	289		288		284	

	Yes	54.8	0.003**	23.2	0.008**	31.1	0.029**

	No	34.2		8.8		16.9	

Can Read English	n	287		286		282	

	Yes	56.5	0.045**	27.5	0.0005***	32.8	0.029**

	No	41.3		12.5		21.5	

^1^ not necessarily by name

^2 ^Weighted to account for variation in sampling probability across facilities.

* 0.1 > p ≥ 0.05

**0.05 > p ≥ 0.001

***p < 0.001

Of the 135 exit interviewees who had heard of an HFC, 80% did not know how members were elected. The few who reported knowing about committee selection mentioned selection through public meetings (n = 25), through meetings organized by the health facility (n = 3), or by Village Health Committees (n = 1).

Exit interviewees were also asked about HFC roles. Of the 135 who knew of HFCs, 48% reported not knowing their roles. Among the 68 who mentioned any role, most commonly noted were administration of the health facility (45%), ensuring drugs and other supplies are available (18%), staff supervision (17%), and ensuring that patients received good services (7%).

A few cases of misunderstanding between HFC members and the wider community were raised in both districts by HFC members, health workers or district managers. For instance, there were reports from both districts about community members making inappropriate demands on the DFF kitty.

*"...once they heard the facility was receiving some money, the community wanted us to make contributions to community projects...they do not understand that the money is used within guidelines..." *(HFC Member, Tana River)

In a related incident, some community members at one facility refused to pay fees at the health facilities claiming there was money to cover all their treatment. The community reportedly did not understand initially that DFF had specific guidelines and could not be spent on other projects or to buy certain items such as drugs.

According to one district manager in Tana River, the community around one dispensary felt dissatisfied with their HFC's performance, forcing the members to resign and electing a new committee. The manager said that it was later discovered that a certain political aspirant had encouraged other community members to force the resignations in order for them to be elected to the committee to further their political ambitions. At another facility in Kwale, HFC members said that those who had not been re-elected to the committee had spread rumours about the remaining members, with the aim of creating a rift between the community and the re-elected members.

In one case, HFC members reportedly complained that community members were suspicious over their handling of facility funds.

*"...The treasurer resigned...he was fed up with the rumours that money was being 'eaten'..." *(Health Worker, Kwale)

FMNs played a significant role in resolving any conflicts that arose within facilities, according to DHMTs. One district manager in Tana River felt that the FMNs were not only crucial to DFF implementation but also to the general coordination of rural health facilities and recommended that the post be introduced throughout the country.

## Discussion

We used a mix of quantitative and qualitative data to investigate the functioning of HFCs as a community accountability mechanism, drawing on the views of health workers, HFC members, patients and district level managers. We found that in a context where HFCs have control over some facility resources through DFF, and receive some training and supervision, they are generally functioning well in both sampled districts (though there were some problems, discussed below). Committees were in place in all facilities and meeting regularly. Selection of members had generally followed DFF guidelines, allowing community involvement and ensuring at least one female per committee. Members usually had good relationships with health workers, and were perceived to play an important role in representing community interests and helping oversee facility management. There were also indications of true depth of involvement with, for example, committee members and health workers agreeing that HFCs made the final decision about employment of subordinate staff and the use of user fee revenues. It is possible that some respondents aimed to present HFCs in an artificially positive light in order to encourage the continued flow of funds for DFF and health system strengthening in general, and the continued provision of HFC allowances! However, problems were also freely raised by respondents, suggesting that this type of bias may be limited.

Two factors were perceived to have facilitated these positive findings. First, FMNs who formed part of the DHMT had specific responsibility for supporting committees and for links between the community and the health service more generally. Their role appeared to have been very important in guiding HFCs in the early stages and in resolving any disputes that arose. Secondly, the introduction of DFF was viewed as having played a crucial role in empowering HFCs to meet, plan and implement activities, increasing their sense of ownership and motivation, and in many facilities transforming committees from dormant entities to active stakeholders. DFF both provided resources which the HFC could manage, and freed up user fee revenues which could then be used to pay allowances to committee members. Stakeholders indicated that without these allowances such high levels of HFC activity would have been very unlikely.

These findings are quite likely to be representative of Coast Province in general, given there was considerable variation between the two sampled districts in terms of accessibility, infrastructure, cultural practices, levels of poverty, and perceived ease of DFF implementation. However, it is unlikely that the results can be generalised to other provinces in Kenya, which had not benefited from FMNs and DFF, though the latter is scheduled to be introduced nationwide under the Health Sector Services Fund from late 2010. In addition, since the early 2000s, Coast Province had benefited from the broader DANIDA-funded programme of health system strengthening. This included training of health workers and managerial staff, construction and renovation, provision of equipment and supplies, strengthening the drug delivery system, enhancing community health activities (including seed funds for income generating activities and strengthening of HFCs), and strengthening of health management information systems (HMIS) and financial management [13]. While some other provinces have also received donor support, it has generally not been this comprehensive.

A number of important limitations were also noted in HFC operation. First, many respondents reported insufficient clarity in HFC roles, with variation in views across stakeholders on the extent to which they could exercise their powers. For example during the card exercise on HFC roles many different views were expressed on the appropriate depth of their involvement in decisions on how to spend DFF funds and in setting user fees. In both scenarios, there were lengthy discussions and in several cases an inability to agree on the HFC role. Lack of clarity in roles, including depth of involvement, has been shown to be an important challenge in other settings (for example Gilson and Erasmus 2006; Lowensen et al., 2004; Khumalo, 2001; cited in Molyneux et al.: Community accountability at peripheral health facilities, submitted). In our case, this lack of clarity may have been exacerbated by the brevity of training, the training of committee officials only in Kwale, and the lack of clear documentation on DFF at a facility level.

Lack of clarity on roles can have important negative implications for the relationship between HFCs and health facility in-charges, a relationship that is crucial to the sustained involvement of committees [5,17] and ultimately their potential for positive influence on health facility performance. While relations were generally good, there were some indications in our study of tensions and mis-trust between health workers and other HFC members. However, to some degree this may be inevitable if HFC members are performing their oversight role appropriately.

As has been noted elsewhere [18,19], there were concerns about the ability of some less educated members to fulfil the more technical roles such as budgeting. With the nationwide scale up of DFF, new guidelines have been gazetted for HFCs which restrict the number of community members to 5, one of whom must have knowledge and experience in finance and administration and three of whom should be women [20]. The rest of the HFC is to be made up of nominated members (the facility in-charge, a representative from the provincial administration, the District Medical Officer for Health or their representative, and the Area Councillor). The implications of these changes for effective community accountability are unclear. While greater education may improve capacity for technical roles, nominated members may inhibit community members from contributing confidently to discussions, and more highly educated community members may be less accessible to some groups of the general population. If the target number of women on HFCs is achieved in future, this may increase the prominence of issues relevant to women and children. However, as observed during our HFC group discussions, the presence of women on committees does not necessarily mean that they will speak freely in front of male members, especially in communities where this is not viewed as a traditionally female role.

HFCs are only one of the mechanisms used to enhance community accountability and engagement in Kenya, with others including public meetings, and the Government's Community Strategy, which aims to deliver services at the community level through community health workers and village health committees. However, it remains a concern that links between the HFC and the broader community are currently so inadequate. Although HFC members were clear that one of their roles was to represent the broader community in health facility operations, less than half of facility users had ever heard of the HFCs. Fewer still knew the chair or any other member of the committee, even if not by name. Women and less well-educated respondents were particularly unlikely to know about the HFC or know its members. Moreover, we only interviewed community members who were using the health facilities, and it is likely that those not using the facility were even less likely to be aware of HFC operations. Engagement by HFCs of the broader community at any depth - even simple information sharing - therefore remains weak. There were also a few reports of poor relationships between committee members and the wider community, including some lack of trust by community members in HFC members' handling of finances.

Strengthened trust and communication is likely to require greater transparency and clarity in HFC selection, roles and activities and on facility finances. Mechanisms to enhance this might include additional public meetings, disseminating information through CHWs or the use of public noticeboards at facilities. Noticeboards (or blackboards) had in fact been supplied to all Coast Province facilities, and at the time of data collection were clearly visible to clients in 25 of the 30 facilities. Facility staff were supposed to complete the blackboard table with monthly data on health and utilization (for example, number vaccinated, under-weight, births and deaths, etc), and accounts (income, expenditure, cash in hand, cash in bank). However, the current utility of these boards was unclear as very few were fully completed, it was unclear whether community members could interpret some of utilization data, and the financial information was limited to bank account totals with no information on how facility funds had been spent [16]. The boards might have been more useful in supporting community engagement if they had instead included the names of all committee members, a brief description of HFC roles, user fee charges, facility income from each source, and expenditure by category for each quarter.

## Conclusions

There is potential for HFCs to function well and play an important role in some aspects of facility management, particularly in a context where funds are available that can be used flexibly, albeit within centrally defined guidelines. This motivates HFCs to meet and to contribute local perspectives to decision-making for the facility.

For the potential of HFCs to be met fully, it is essential that all actors are clear about the roles of HFCs, how committee members are selected, and - most fundamentally - the extent and limits of their powers in different areas. In our setting the key areas requiring greater clarity were depth of involvement in decisions over spending DFF money, and over increasing user fee charges; both of which are important areas in ensuring that health services are locally acceptable and accessible. Improved clarity in roles in turn requires strong training, documentation and supportive supervision from the district. Failure in any or all of these areas can undermine effectiveness of operations and contribute to conflicts.

In addition, HFCs should be representatives of a broader community, requiring appropriate selection from and links to those communities. It is essential that mechanisms for communicating with the wider community are carefully considered when establishing HFCs, and maintained over time, to ensure that accountability to the broader population is achieved.

## Competing interests

The authors declare that they have no competing interests.

## Authors' contributions

CG and SM conceptualized the study, participated in data collection, interpretation of results and drafting of the paper. AO designed study tools, participated in data collection, analysis and drafting of the paper. MK participated in data collection and analysis of the results. All authors read and approved the manuscript.

## Pre-publication history

The pre-publication history for this paper can be accessed here:

http://www.biomedcentral.com/1472-6963/11/229/prepub
